# Woman With Lethargy and Bradycardia

**DOI:** 10.1016/j.acepjo.2025.100082

**Published:** 2025-03-05

**Authors:** Eshwar Kishore, Ray Fowler, Brian Miller

**Affiliations:** Department of Emergency Medicine, University of Texas Southwestern Medical Center, Dallas, Texas, USA

**Keywords:** bradycardia, prehospital, EKG, electrocardiogram, hyperkalemia, pacemaker, ESRD

## 1 Patient Presentation

**Figure 1:**
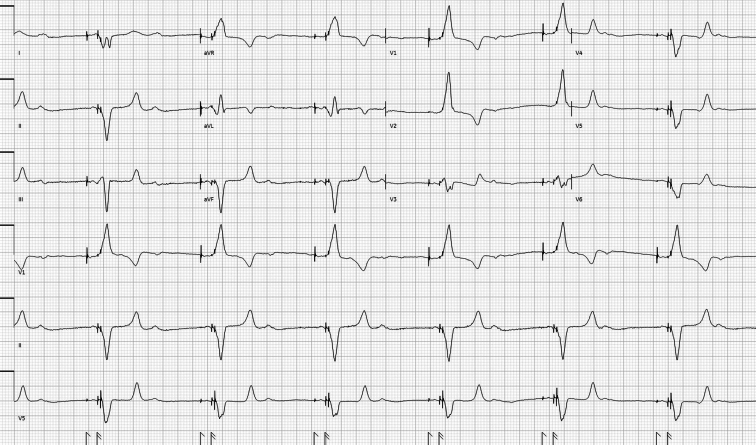
Initial electrocardiogram obtained on emergency department presentation - bradycardic atrioventricular dual-paced rhythm with ventricular rate of 39 counts per minute.

A 59-year-old woman with a history of hypertension, end-stage renal disease on peritoneal dialysis (PD), and heart failure with reduced ejection fraction of 20% status post cardiac resynchronization therapy with a defibrillator presented to the emergency department with an acute onset of profound generalized weakness and lethargy that had developed over the last hour. She was found to be bradycardic to the upper 30s, with an elevated blood pressure. Her family reported that she had skipped her last intermittent PD session. An initial electrocardiogram (EKG) was obtained (Fig. 1). Intravenous calcium was administered empirically for hyperkalemia, and laboratory testing confirmed potassium was 8.3 mmol/L. Nephrology was consulted to perform an emergent manual PD exchange followed by rapid cycling PD over hours. Her bradycardia progressively resolved as her potassium levels improved to 5.4 mmol/L.

## 2 Diagnosis: Oversensing and Ventricular Pacing Inhibition Resulting in Symptomatic Bradycardia

**Figure 2:**
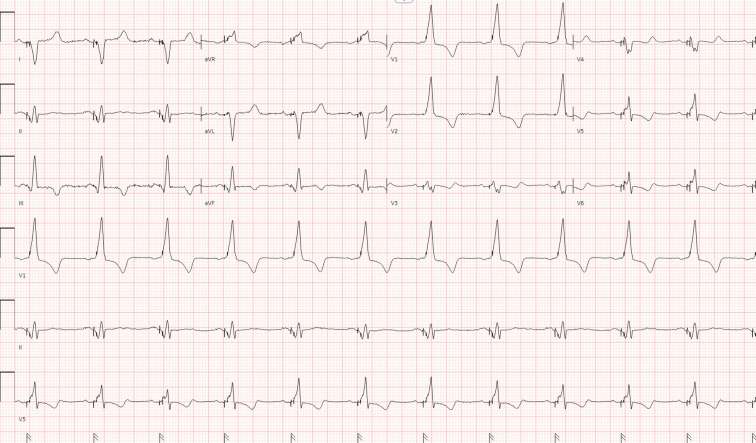
Repeat electrocardiogram obtained after correction of hyperkalemia - atrioventricular dual-paced rhythm with ventricular rate of 68 counts per minute.

Oversensing occurs when a pacemaker inappropriately interprets electrical signals as intrinsic cardiac activity and inhibits pacer stimuli.^1^ Causes can include electromagnetic interference, pacemaker lead failure, and skeletal muscle contractions. In this patient's case, missed PD led to acute hyperkalemia, which triggered oversensing and ventricular pacing inhibition, resulting in symptomatic bradycardia.^2^ Hyperkalemia manifests in multiple electrocardiographic abnormalities, characterized by peaked T-waves, prolonged PR intervals with P-wave loss, and progressive QRS complex widening (PR interval is the time from the onset of the P wave to the start of the QRS complex, and the QRS complex are graphical deflections that show the depolarization/contraction of the ventricles).^3^ These abnormalities are demonstrated in the patient’s initial EKG (Fig. 1) with loss of P-waves and large amplitude peaked T-waves being misinterpreted as QRS complexes by the pacemaker.^1^ A repeat EKG (Fig. 2) after hyperkalemia correction showed appropriate function of pacemaker.

## Funding and Support

By *JACEP Open* policy, all authors are required to disclose any and all commercial, financial, and other relationships in any way related to the subject of this article as per ICMJE conflict of interest guidelines (see www.icmje.org). The authors have stated that no such relationships exist.
